# Websites Selling Direct-to-Consumer Anti-Mullerian Hormone Tests

**DOI:** 10.1001/jamanetworkopen.2023.30192

**Published:** 2023-08-21

**Authors:** Alexis Johnson, Rachel Thompson, Brooke Nickel, Patti Shih, Karin Hammarberg, Tessa Copp

**Affiliations:** 1 Faculty of Medicine and Health, School of Public Health, The University of Sydney, Sydney, New South Wales, Australia; 2 Faculty of Medicine and Health, School of Health Sciences, The University of Sydney, Sydney, New South Wales, Australia; 3 Faculty of Arts, Social Sciences and Humanities, School of Health and Society, Australian Centre for Health Engagement, Evidence and Values, University of Wollongong, Wollongong, New South Wales, Australia; 4 School of Public Health and Preventive Medicine, Monash University, Melbourne, Victoria, Australia

## Abstract

**Importance:**

The recent provision of direct-to-consumer (DTC) Anti-Mullerian Hormone (AMH) testing in several countries has been contentious, particularly due to concerns about judicious testing and informed consent.

**Objective:**

To describe and analyze information on websites that sell DTC AMH tests.

**Design, Setting, and Participants:**

Qualitative study including content analysis of text information from websites in multiple countries that sell AMH tests DTC. The top 50 search results from 4 different internet search strings were captured and reviewed for eligibility. Data were extracted in March 2022 and analyzed from April 2022 to July 2023.

**Main outcomes and measures:**

Themes and categories were derived from the website content using a conventional inductive approach, with a particular focus on information content, quality and accuracy, as well as the tone and language used.

**Results:**

Twenty-seven websites across 7 different countries formed the sample for analysis. Information varied considerably across websites and was organized into 6 overarching categories: (1) whether a test description was included (25 websites [93%]); (2) statements about what the test can do, which included indicating ovarian reserve (26 websites [96%]) and indicating likelihood of conceiving (20 websites [74%]); (3) statements about the usefulness of the test result, which included enabling women to adjust their reproductive timeline (11 websites [41%]) and determining whether egg freezing was a viable option (8 websites [30%]); (4) blood collection method (ie, through a laboratory or an at-home sample); (5) promotion tactics, such as stating the convenience of testing (24 websites [89%]) and using language promoting empowerment and control (7 websites [26%]); and (6) statements about limitations of the test, which included polycystic ovary syndrome falsely inflating AMH levels (13 websites [48%]) and that it cannot accurately predict chances of conceiving (9 websites [33%]).

**Conclusion and relevance:**

In this qualitative study including content analysis, most websites selling DTC AMH tests included false and misleading claims which might lead consumers to purchase an AMH test in the belief that it can reliably predict fertility potential and age of menopause. Depending on the test result, this may in turn lead to misplaced anxiety or reassurance about one’s fertility and modifications to subsequent conception or contraceptive plans and behavior.

## Key Points

**Question:**

What information is presented by companies selling the anti-Mullerian hormone (AMH) test direct to consumers online?

**Findings:**

In this qualitative study including content analysis of 27 websites across multiple countries, a content analysis showed that the amount and type of information was highly varied. Most contained statements about the AMH test that are not supported by evidence, including that it can reliably predict fertility potential or age of menopause.

**Meaning:**

These findings suggest false claims about the AMH test are common, misleading consumers to purchase an AMH test in the belief that it can predict current or future fertility, which may lead to misplaced anxiety or reassurance about one's fertility.

## Teaser

This qualitative study including content analysis evaluates information presented on websites by companies across the globe selling direct-to-consumer anti-Mullerian hormone tests.

## Introduction

The average age of mothers at first birth is increasing in high-income countries,^1^ in part explained by the rise of effective contraception, increases in women’s education and labor market participation, value changes, gender equity, and partnership changes.^2^ As female fertility decreases with advancing age, this increases the risk and prevalence of involuntary childlessness.^3^ Over the past decade, there has been growing attention in both the media and in academic literature around the anti-Mullerian hormone (AMH) test (often nicknamed the egg timer test),^4,5^ with some clinicians and researchers suggesting routine screening to increase reproductive awareness and the chance of parenthood in women with accelerated ovarian aging.^6^ AMH is produced by the granulosa cells in the ovarian follicles.^7^ AMH levels in the blood correspond to the number of antral follicles in the ovaries and is used to estimate ovarian reserve.^8,9^ Although AMH testing is useful in the context of fertility treatment, as it indicates the number of eggs that can be retrieved in a stimulated cycle for in vitro fertilization (IVF) or egg freezing,^10^ AMH is not a reliable measure of fertility. However, recent introduction of direct-to-consumer (DTC) AMH testing in several countries^4,11,12^ has expanded public access to the test and provided a pathway to testing without the involvement of or counseling from an independent health care practitioner.

There are several limitations to the AMH test. Although it is associated with the number of eggs in the ovaries, evidence consistently suggests AMH levels are a poor predictor of current and future fertility for an individual woman,^13,14,15^ in part because they do not provide any indication of egg quality^16^ nor how quickly a woman’s egg count is declining. For example, a recent time-to-pregnancy cohort study^14^ of women aged 30 to 44 years without a history of infertility found that there was no significant association between diminished ovarian reserve (AMH level <0.7 ng/ml) and risk of future infertility (relative risk [RR], 0.65; 95% CI, 0.21-2.07) or future fecundability (fecundability ratio, 0.97; 95% CI, 0.59-1.60). In addition, while AMH levels are associated with age of menopause at a population level,^17,18^ age predictions have a broad age range, making them of limited practical utility for individual women.^19,20^ In addition, it is currently unclear whether repeated testing provides a more accurate indication of egg count decline.^21^ Due to these limitations, professional society guidance in the US currently discourages AMH testing in the general population.^22,23^

In the context of DTC testing, consumers’ ability to exercise autonomy and provide informed consent rests entirely on the quality and comprehensiveness of the information provided by the test vendor. For other DTC tests, significant limitations in information provision have been identified.^24,25^ In addition, in a previous study, we identified several false or misleading claims about the utility and benefits of the AMH test on fertility clinic websites in Australia and New Zealand.^26^ The quality and comprehensiveness of information provided on websites that offer DTC AMH testing is currently unknown. Therefore, the aim of this qualitative study including content analysis was to describe and analyze information about the AMH test that is provided on websites that sell AMH tests DTC, with a particular focus on information content, the quality and accuracy, as well as the tone and language used.

## Methods

### Study Design

We conducted a content analysis of text on websites that sell AMH tests DTC. Content analysis is a widely used qualitative research technique, which also uses quantitative methods to analyze written content, enabling themes, meanings, and concepts to be quantified and evaluated through coding.^27^ As this study aimed to describe a phenomenon (the written information on websites selling AMH tests DTC), we undertook a conventional, inductive approach to content analysis.^28^ This approach derives categories from the data without imposing preconceived theories or categories.^28^ Ethics approval was not required for this research as it did not involve human participants and all data were derived from publicly available information online. The study is reported according to the Standards for Reporting Qualitative Research (SRQR) reporting guideline.^29^

### Eligibility Criteria

English-language websites originating in any country where consumers can purchase an AMH test directly (ie, contained an add to cart option) were included. Fertility clinic websites (without a DTC add to cart option), websites providing information on the AMH test but not selling the test, and websites that offered fertility testing but not AMH tests specifically (ie, tested follicle-stimulating hormone [FSH] only) were excluded.

### Search Strategy

We used the Google search engine in a Google Chrome browser to search for eligible websites. Before conducting the search, private browsing was enabled, and the browser cache and cookies were cleared to minimize personalization of results. Four search strings were used: (1) *AMH test online*, (2) *Where can I order an AMH test online*, (3) *Ovarian reserve test online*, and (4) *Buy test of my reproductive timeline online*. The top 50 results for each search string were extracted into an excel spreadsheet, with duplicate and ineligible websites removed. Very few nonduplicate websites were identified by the fourth search string, providing a level of confidence that relevant websites were captured. Searches were conducted and data extracted in March 2022.

### Statistical Analysis

Two researchers (A.J. and T.C.) independently reviewed information about the AMH test across all the captured websites to develop an initial list of reoccurring codes and themes, in addition to recording website characteristics (country, cost of test, and so forth). These lists were then integrated and organized into broader categories to form the initial coding framework, which was refined and discussed with 2 additional researchers (R.T. and B.N.). Each website was then coded using the framework by 2 researchers independently (A.J. and T.C.), with 8 websites (30%) double-coded to confirm consistency in application of the framework. The level of agreement between the 2 coders was tested using Cohen κ and indicated a strong level of agreement (κ = 0.74).^30^ Any inconsistencies between the coders were resolved through discussion with the broader study team to reach consensus. As with all qualitative research, the identified codes and themes may have been shaped by the coders’ personal characteristics and research interests.^29^ The 2 coders (A.J. and T.C.) were female researchers of reproductive age, who do not have children but would like to have children in the future. A.J. was a Master of Public Health student conducting this study in requirement of her degree. T.C. was a public health researcher with a focus on women’s reproductive health and evidence-based medicine, and has extensive qualitative experience. Data were analyzed in SPSS version 28 (IBM) from April 2022 to July 2023.

## Results

### Included Websites

Of the 200 websites extracted following implementation of the search strategy, 98 did not discuss the AMH test, and a further 35 were about the AMH test but did not sell the test DTC. Of the remaining 67 websites, 37 were duplicates, leaving 30 unique websites for data extraction. During analysis, a further 3 websites were excluded (1 was undergoing maintenance, 1 tested FSH only, and 1 did not sell the AMH test DTC), leaving 27 eligible websites.

### Website Characteristics

Table 1 displays characteristics of the websites. Websites originated from the US (9 websites [33%]), India (6 websites, [22%]), the United Kingdom (4 websites [15%]), Australia (3 websites [11%]), Canada (2 websites [7%]), Ireland (2 websites [7%]), and the Netherlands (1 website [4%]). Most websites offered the AMH test alone (23 websites [85%]), although 4 websites tested multiple hormones (eg, AMH, FSH, and estradiol E2 combined in 1 test). The cost of the tests varied substantially, ranging from approximately US $16 to $214.

**Table 1.** Characteristics of Included Websites

Company	Country	Test cost, US$^a^	Concurrently tests hormones other than AMH	Specified an age requirement for testing	Includes option of physician consult after test	Provided a graph or table of results by age	Provided consumer testimonials or reviews	Included implicit or explicit endorsement^b^
Access Labs	US	122	No	No	No	No	Yes	No
Apollo Diagnostics	India	26	No	No	No	No	No	No
ASDA by ZAVA	United Kingdom	90	No	No	No	No	No	Yes
Blue Horizon Medicals	Canada	130	No	No	No	No	No	No
Care on Call	Ireland	129	No	No	No	No	No	No
Diagnostic Centres	India	17	No	No	No	No	No	No
DxSaver	US	98 lab test; 143 home kit	No	No	No	Yes	Yes	No
Hey Grip	Netherlands	201	Yes	No	Yes	No	Yes	Yes
I Screen	Australia	75	No	No	No	No	No	No
I Medical	Australia	70	No	No	No	No	No	No
Kin Fertility	Australia	212	Yes	Yes	Yes	No	Yes	Yes
Labcorp	US	183	Yes	No	No	No	No	No
LifeLabs	Canada	55	No	No	No	No	No	No
Medichecks	United Kingdom	99	No	No	Yes	No	Yes	Yes
MediFee	India	16	No	No	No	No	No	Yes
Metropolis	India	28	No	No	No	Yes	Yes	Yes
Modern Fertility	US	163	Yes	Yes	No	No	Yes	Yes
Request a Test	US	102	No	No	No	No	No	No
Superdrug	United Kingdom	97	No	No	Yes	Yes	No	Yes
Tata 1mg	India	16	No	No	No	Yes	No	Yes
Testing.com	US	142	No	No	No	No	No	Yes
Thyrocare	India	17	No	No	No	No	Yes	
Tree of Life Fertility	US	102	No	No	Yes	No	No	Yes
Unilab	US	204	No	No	No	No	No	No
Walk-in Lab	US	96	No	No	No	No	No	No
Webdoctor	Ireland	128	No	No	No	No	No	No
ZAVA	United Kingdom	87	No	No	No	No	No	Yes

Abbreviation: AMH, anti-Mullerian hormone.

^a^ All currencies were converted to US dollars using the OANDA Currency Converter in May 2022.^45^

^b^ Implicit or explicit endorsement included statements such as “medically reviewed,” or including images of physicians in white lab coats.

### Content Analysis Findings

Information about the AMH test was organized under 6 overarching categories: (1) description of the test, (2) statements about what the test can do (test utility), (3) stated possible actions or decisions based on the test result (next steps), (4) collection method, (5) promotional tactics, and (6) statements about the test’s limitations. All categories and their associated subcategories are shown in Table 2, with the frequency and an example quote.

**Table 2.** Statements About the AMH Test on Direct-to-Consumer Websites

Categories and subcategories	Example quote	No. (%)
Brief description of test	“It is a blood test that measures the Anti-Mullerian Hormone, a specific hormone produced by the ovaries.” (Care on Call)	25 (93)
Statements about what the test can do		26 (96)
Assesses number of eggs/ovarian reserve	“Decreased AMH levels indicate low quality and number of eggs.” (Diagnostic Centres)	26 (96)
Assesses fertility/likelihood of conceiving^a^	“AMH (anti-mullerian hormone) is a female hormone that is important for measuring fertility levels.” (ASDA by ZAVA)	20 (74)
Indicates menopause timing^a^	“It can also be helpful in predicting the onset of menopause.” (iMedical)	20 (74)
Indicates diminished ovarian reserve/early menopause^a^	“We’ll also tell you if you’re at risk of an early menopause.” (HeyGrip)	11 (41)
Assists with ART/predicts outcomes of IVF	“Increased AMH may indicate an increased or even excessive responsiveness to IVF and a need to tailor the procedure accordingly.” (Testing.com)	19 (70)
Indicates/confirms PCOS	“It can also indicate conditions like PCOS (polycystic ovarian syndrome) which may affect your ability to conceive.” (Labcorp)	26 (96)
Tumor detection/effect of cancer treatment	“Evaluating the effectiveness of the treatment of ovarian cancer is also another major purpose of it.” (Diagnostic Centres)	10 (37)
Delayed puberty for boys	“AMH has also been studied in conjunction with the measurement of FSH LH and testosterone for precocious and delayed puberty in boys.” (Apollo Diagnostics)	4 (15)
Reason for lack of menstruation	“Help to find the reason for lack of menstruation, amenorrhea.” (Walk-In Lab)	5 (19)
Stated actions or decisions based on test result		13 (48)
Helps with family planning and indicates whether you need to adjust your reproductive timeline	“An AMH test may help a woman better understand her fertility, allowing her to make informed decisions about conceiving in the future should she so wish.” (Care on Call)	11 (41)
Enables you to address a potential problem while there is still time	“If your levels are lower than expected for your age, you may want to visit a specialist fertility clinic to discuss what options are available to you.” (Superdrug)	5 (19)
Helps to determine if egg freezing viable	“As an ovarian reserve test, the AMH blood test can help assess the egg supply to guide IVF efforts and egg freezing decisions.” (Access Labs)	8 (30)
Enables you to treat low AMH levels (eg, vitamins, stress reduction)	“The low levels of AMH can be treated with the following medications and therapies. Vitamin D supplements, L-arginine supplementation, Abdominal massages to increase the blood circulation to the ovaries, Stress reduction techniques like yoga and meditation.” (Dx Saver)	2 (7)
Advises repeated testing every few years	“An AMH test cannot tell you how good your eggs are or how quickly you are losing your follicles. You may need to do more than one AMH test or do other tests to get a clearer picture of your fertility.” (ZAVA)	3 (11)
Collection method		26 (96)
Take at home/finger prick	“A small sample of blood is collected via a simple finger prick and sent off to the accredited laboratory used by careoncall..” (Care On Call)	14 (52)
Go to laboratory/collection center	“Get your blood sample collected at our partner pathology centres all around Australia.” (Kin Fertility)	16 (59)
Promotion tactics		25 (93)
Included a discount or code	“Rs. 2000.00 1200.00 (Online 45% Discount).” (MediFee)	5 (19)
Language suggesting empowerment and control	“My body, my data, my choice.” (HeyGrip); “Be proactive about your fertility.” (Modern Fertility)	7 (26)
Emphasizes convenience	“The only comprehensive fertility hormone test you can take in your jammies.” (Modern Fertility)	24 (89)
Included statistics of the prevalence of infertility or premature menopause	“Why does it matter? About 1 in 100 people suffer from early ovarian failure, which basically means you enter menopause before 45. The hormone AMH correlates with the number of follicles that you have left in your ovarian reserve, and is the most reliable predictor that we’ve got.” (HeyGrip)	6 (22)
Included an explicit statement that the test was suitable for a wide range of women, irrespective of attempts to conceive	“Whether you’re years away from kids or thinking of trying soon, we’ll guide you through your fertility hormones now so you have options later.” (Kin Fertility)	10 (37)
Stated limitations about the test		19 (70)
Not appropriate if using OCPs	“Note this test is not appropriate for individuals taking the oral contraceptive pill.” (IScreen)	7 (26)
PCOS falsely inflates AMH	“Women with polycystic ovaries can have higher than expected results for their age.” (Medichecks)	13 (48)
AMH is not predictive of chance of conceiving	“This test can’t tell you your exact chances of falling pregnant. Or whether you are infertile. There is no sure-fire test that can. This Fertility Hormone test is the best first step in understanding your fertility proactively.” (Kin Fertility)	9 (33)
Certain factors influence AMH levels	“Research indicates that in black and Hispanic women, serum AMH levels can be 25% lower than those found in Caucasian women of a similar age.” (Webdoctor)	6 (22)

Abbreviations: AMH, anti-Mullerian hormone; ART, assisted reproductive technology; FSH, follicle-stimulating hormone; IVF, in vitro fertilization; LH, luteinizing hormone; OCP, oral contraceptive pill; PCOS, polycystic ovary syndrome.

^a^ Statements of utility not supported by the current evidence.

### Test Description

Most websites provided a brief description of the test (25 websites [93%]). Commonly, this description explained that the AMH test was a blood test measuring AMH, a hormone produced by the ovaries.

### Test Utility

Most websites included statements about what the test can do (26 websites [96%]). These were that the test can assess the number of eggs or ovarian reserve (26 websites [96%]), indicate or confirm polycystic ovary syndrome (PCOS) (26 websites [96%]), estimate fertility and the likelihood of conceiving (20 websites [74%]), indicate menopause timing (20 websites [74%]), indicate a diminished ovarian reserve or early menopause (11 websites [41%]), detect tumors or examine the effect of ovarian cancer treatment (10 websites [37%]), and help find a reason for a lack of menstruation (5 websites [19%]) or a reason for delayed puberty in boys (4 websites [15%]).

### Next Steps

Some websites stated actions or decisions consumers could make based on the test result (13 websites [48%]). The most common statements were that the test result enables women to be proactive about their fertility and adjust their reproductive timeline (11 websites [41%]), helps determine whether egg freezing is a viable option (8 websites [30%]), and enables women to address any red flags about their fertility while there is still time (5 websites [19%]). Three websites recommended repeated testing every few years (3 websites [11%]), and 2 websites suggested low AMH levels could be treated, for example, through stress reduction or vitamins (2 websites [7%]).

### Test Collection Method

Almost all websites (26 websites [96%]) stated how the blood test could be collected, with only 1 website not disclosing how the testing was undertaken. There were 2 different methods, with a few websites offering both: going to a laboratory or collection center for blood collection (16 websites [59%]) or through a finger prick at-home sample (14 websites [52%]).

### Promotional Tactics

Most websites used promotional tactics (25 websites [93%]), or language portraying the test in a positive light, to help entice a buyer for their product. The most common strategy was emphasizing the ease and convenience of the test, such as it being a “simple finger prick test” that can be taken any day of the menstrual cycle with no referral required (24 websites [89%]); followed by including explicit statements about who the test was suitable for, irrespective of attempts to conceive (10 websites [37%]); using language promoting empowerment, control, and being proactive, such as “power in fertility info” (7 websites [26%]); including statistics regarding the prevalence of infertility or premature menopause, which may potentially increase the perceived need for testing (6 websites [22%]); or offering a discount code (5 websites [19%]).

### Test Limitations

Several websites stated a limitation of the AMH test (19 websites [70%]). These included that having PCOS can falsely inflate AMH levels (13 websites [48%]), that an AMH test cannot predict a woman’s chances of conceiving (9 websites [33%]), that the test is not reliable when taking the oral contraceptive pill (OCP) (7 websites [26%]), and that certain factors (eg, ethnicity, obesity, or smoking) can influence AMH levels (6 websites [22%]).

## Discussion

This study found many misleading claims about the value of the AMH test on websites that sell DTC AMH tests. This increases the risk of women having to rely on nonevidence-based information in their reproductive decision-making. The amount of information provided was also highly varied, ranging from an option to purchase the test with no accompanying information, through to detailed information about what the test can do and actions consumers can take based on the result.

Concerningly, three-quarters of the websites stated that the test was a way of gaining insight into one’s fertility and chances of conception, despite evidence showing the test cannot reliably predict current or future fertility.^13,14^ In addition, the majority of websites claimed that the test could indicate menopause timing or identify women at risk of premature menopause, despite the considerable imprecision in such estimates.^17,20^ Considering the lack of evidence about the utility of the test, statements suggesting the result of the test can inform reproductive timelines are deceptive. Two websites claimed low AMH levels could be treated, such as with vitamin D supplementation or stress reduction, despite little evidence to support such a claim.^31,32^ Conflicting with the American Congress of Obstetricians and Gynaecologists guidance,^22^ DTC websites target a wide range of consumers of reproductive age and not only those actively trying to conceive (eg, “we encourage women of all ages to check their AMH”), possibly to broaden their market and increase revenue.^33^ Although some websites acknowledged important limitations of the AMH test, more often these were not mentioned or contradicted. For example, while some websites correctly noted the test result could be unreliable when using OCP,^34^ others either did not disclose this or actively encouraged use of the test among consumers using OCP (eg, “you’ll test AMH even on birth control”).

Although all websites were promoting the test to consumers, some used persuasive or notable marketing techniques to actively encourage test use, such as including physician testimonials or emphasizing the simplicity and convenience of testing. Most notably, some websites used feminist arguments or framing, including language such as “be proactive” or “empower your health” to encourage test uptake and evoke the notion that the test helps women take control of their fertility. The use of the feminism argument has been seen before in the promotion of other medical interventions or treatments with limited evidence, such as in the lobbying for flibanserin for low sexual desire.^35^ Ironically, deciding to get an AMH test based on misleading information is the opposite of empowering and undermines informed decision-making.^36^

The identified problems with the poor quality of information on the websites included in this study have significant implications for consumers’ ability to exercise autonomy and provide informed consent to the AMH test. We see 2 major negative consequences of this. First, consumers may be falsely reassured by test findings and as a result delay plans to conceive, despite age being the most important factor influencing fertility. Second, consumers may be needlessly concerned if they receive a low test result, fearing this suggests subfertility or possible future problems conceiving. This may pressure women to conceive earlier than desired or seek potentially unnecessary fertility treatments, such as IVF or egg freezing.^37,38^ Inaccurate information also has implications for health professionals, who may be required to help women interpret their results. Websites that include misleading statements or do not state the test’s limitations are placing a large burden on clinicians to dispel misconceptions, which can be very time consuming.^36^

Given the increasing trend to delay childbearing and subsequent increase in age-related infertility, some proponents of the test suggest universal AMH screening may prompt women to prioritize conception before significant age-related fertility decline.^6,39,40^ However, the lack of an association between AMH and current^13^ and future fertility^14^ means this can be misleading. Efforts to increase fertility knowledge (eg, public and education-based fertility awareness campaigns and greater attention to reproductive health in routine health care) in combination with efforts to address the structural barriers to earlier childbearing are likely to be more effective in enabling people to achieve their reproductive aspirations.^2,41^ The growing interest among health professionals in reproductive health education and fertility health promotion^42^ may result in the implementation of such strategies. Public education about the lack of utility of AMH testing for women not undergoing infertility treatment is also needed to prevent women undergoing testing in the belief that it can provide reliable insights into their fertility and reproductive timeline.^43^

Although regulation for product safety, quality, and efficacy exists in some countries, DTC test marketing and advertising is minimally regulated.^33^ For example, even when regulation about the advertising of testing exists, there are several challenges. In Australia, the Therapeutic Goods Administration regulates advertising of therapeutic goods, but it relies on consumers and physicians submitting complaints, and the extent of investigation and follow-up are often brief. In addition, most regulation concerning DTC tests falls within in-vitro devices regulation, which focuses on safety, efficacy, and quality of the tests. This only concerns the technical aspect of testing, that is, whether the testing devices and processes used are safe, accurate, and high quality. Regulations do not consider the practice of testing, that is, the wider social and clinical context by which testing decisions are made. Issues of concern which this study raises, such as whether the test is appropriate and beneficial for the specific consumer or whether it is the correct test to achieve the claimed purpose (eg, indicate fertility), can often be in the blind spot. Although future research is needed to explore how different regulatory environments influence the information provided, findings of this study suggest there is an urgent need for better regulation of AMH test marketing and advertising. This is particularly the case as DTC access removes any opportunities for counseling or discussion of the test’s limitations before testing.^44^ There is a need to explore the role and effectiveness of test regulation for preventing or addressing issues identified by this study, including whether more or different legal and regulatory frameworks or modified approaches to compliance monitoring and enforcement would improve information quality and comprehensiveness.

### Limitations and Strengths

This study has some limitations. First, we only assessed written online content and excluded audio-visual content. This method may have potentially missed some more nuanced information about the test’s limitations or other disclaimers; however, only 2 websites contained audio-visual content. Second, our search strategy was performed using an Australian IP address. Searches in different countries would possibly yield different results; however, we were able to still capture websites across 7 different countries through our thorough search strategy. Third, we compared costs converted to US dollars using the OANDA Currency Converter^45^ in May 2022 to enable comparison across the 7 different countries, without adjustment for the variable cost of living and gross domestic products in different countries.

The study also has several strengths and is to our knowledge the first to assess the quality of information on websites that sell AMH tests DTC. A comprehensive search strategy was used to capture a wide range of DTC websites across several countries. Each subsequent search string had fewer unique results than the previous search string with many duplicates, suggesting we captured as many relevant websites as possible. We also used the well-established content analysis method to enable the analysis of text using both quantitative and qualitative methods,^27,28^ with 8 of the websites double-coded (30%) to ensure consistency. Future research should examine how women in the community interpret online information about the AMH test, as well as the results that are provided after testing, including whether counseling about the test’s limitations reduces the risk of misinterpretation.

## Conclusions

In this qualitative study including content analysis, websites across several countries offering DTC AMH testing were easily found through simple internet searches and most contained false and misleading claims about the utility of the AMH test. This has the potential to mislead consumers to purchase an AMH test in the belief that it can predict fertility potential or age of menopause.

## Notes

Supplement.
Data Sharing Statement
