# Supplementary material for: Websites Selling Direct-to-Consumer Anti-Mullerian Hormone Tests
Source: JAMA Netw Open. 2023 Aug 21;6(8):e2330192. doi: 10.1001/jamanetworkopen.2023.30192 (PMC10442712; doi:10.1001/jamanetworkopen.2023.30192)
Supplement: Supplement. — Data Sharing Statement [file jamanetwopen-e2330192-s001.pdf]

Data Sharing Statement

Johnson. Websites Selling Direct-to-Consumer Anti-Mullerian Hormone Tests. *JAMA Netw Open*. Published August 21, 2023. doi:10.1001/jamanetworkopen.2023.30192

Data
Data available: Yes
Data types: Data (not involving human participants)
How to access data: The data were derived from publicly available information on websites selling an AMH test direct-to-consumers. The content analysis coding framework is available upon request (email: tessa.copp@sydney.edu.au)
When available: With publication

Supporting Documents
Document types: None

Additional Information
Who can access the data: The data will be made available to anyone making a reasonable request.
Types of analyses: Any purpose
Mechanisms of data availability: With investigator support
